# 2′-Methyl-3,5-diphenyl­spiro­[4,6-dioxa-2-aza­bicyclo­[3.2.0]hept-2-ene-7,4′-iso­quinoline]-1′,3′(2′*H*,4′*H*)-dione

**DOI:** 10.1107/S1600536810008718

**Published:** 2010-03-13

**Authors:** Hoong-Kun Fun, Jia Hao Goh, Haitao Yu, Yan Zhang

**Affiliations:** a X-ray Crystallography Unit, School of Physics, Universiti Sains Malaysia, 11800 USM, Penang, Malaysia; b School of Chemistry and Chemical Engineering, Nanjing University, Nanjing 210093, People’s Republic of China

## Abstract

In the title compound, C_25_H_18_N_2_O_4_, the tetra­hydro­pyridine ring adopts a distorted envelope conformation with the spiro C atom at the flap position [deviation = 0.470 (2) Å]. The dihydro–oxazole ring is planar (r.m.s. deviation = 0.013 Å) and it makes dihedral angles of 73.43 (8) and 4.24 (8)° with the two attached phenyl rings. The dihedral angle between oxetane and oxazole planes is 67.44 (9)°. In the crystal structure, C—H⋯O hydrogen bonds link neighbouring mol­ecules into zigzag chains along the *b* axis and these chains are linked *via* C—H⋯π inter­actions.

## Related literature

For general background to and applications of isoquinolinedione derivatives, see: Hall *et al.* (1994 ▶); Malamas & Hohman (1994 ▶); Nan *et al.* (2004 ▶). For ring conformations, see: Cremer & Pople (1975 ▶). For related structures, see: Fun *et al.* (2010 ▶); Wang *et al.* (2000 ▶). For bond-length data, see: Allen *et al.* (1987 ▶). For the stability of the temperature controller used for the data collection, see: Cosier & Glazer (1986 ▶).
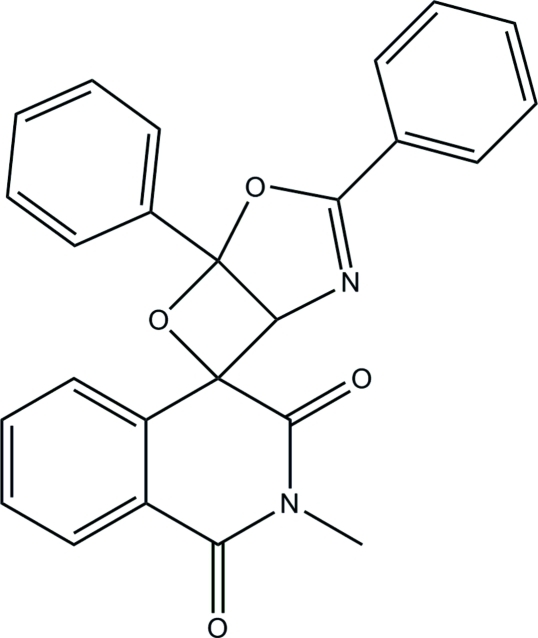


## Experimental

### 

#### Crystal data

C_25_H_18_N_2_O_4_
*M* *_r_* = 410.41
Monoclinic, 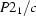

*a* = 13.3142 (3) Å
*b* = 8.0366 (2) Å
*c* = 19.1913 (5) Å
β = 109.882 (1)°
*V* = 1931.09 (8) Å^3^
*Z* = 4
Cu *K*α radiation
μ = 0.79 mm^−1^
*T* = 100 K
0.27 × 0.26 × 0.25 mm

#### Data collection

Bruker SMART APEX DUO CCD area-detector diffractometer
Absorption correction: multi-scan (*SADABS*; Bruker, 2009 ▶) *T*_min_ = 0.815, *T*_max_ = 0.830
32042 measured reflections
3254 independent reflections
3227 reflections with *I* > 2σ(*I*)
*R*_int_ = 0.018

#### Refinement

*R*[*F* ^2^ > 2σ(*F* ^2^)] = 0.047
*wR*(*F* ^2^) = 0.150
*S* = 1.35
3254 reflections
353 parameters
All H-atom parameters refined
Δρ_max_ = 0.80 e Å^−3^
Δρ_min_ = −0.88 e Å^−3^

### 

Data collection: *APEX2* (Bruker, 2009 ▶); cell refinement: *SAINT* (Bruker, 2009 ▶); data reduction: *SAINT*; program(s) used to solve structure: *SHELXTL* (Sheldrick, 2008 ▶); program(s) used to refine structure: *SHELXTL*; molecular graphics: *SHELXTL*; software used to prepare material for publication: *SHELXTL* and *PLATON* (Spek, 2009 ▶).

## Supplementary Material

Crystal structure: contains datablocks global, I. DOI: 10.1107/S1600536810008718/ci5051sup1.cif

Structure factors: contains datablocks I. DOI: 10.1107/S1600536810008718/ci5051Isup2.hkl

Additional supplementary materials:  crystallographic information; 3D view; checkCIF report

## Figures and Tables

**Table 1:** Hydrogen-bond geometry (Å, °) *Cg*1 is the centroid of the C13–C18 benzene ring.

*D*—H⋯*A*	*D*—H	H⋯*A*	*D*⋯*A*	*D*—H⋯*A*
C17—H17*A*⋯O1^i^	0.97 (2)	2.51 (2)	3.213 (2)	129 (1)
C23—H23*A*⋯*Cg*1^ii^	0.96 (2)	2.66 (2)	3.5904 (18)	166 (2)

Symmetry codes: (i) 
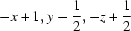
; (ii) 
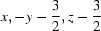
.

**Fig. 1.**
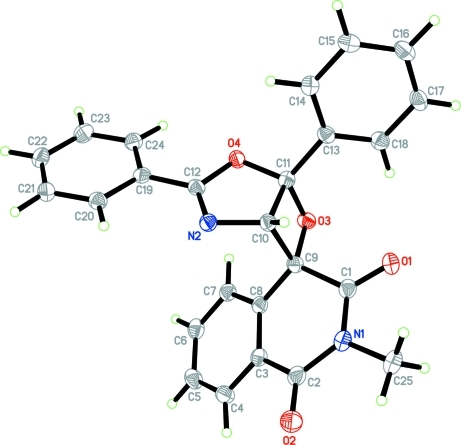
The molecular structure of the title compound, showing the atomic numbering scheme. Displacement ellipsoids are drawn at the 50% probability level.

**Fig. 2.**
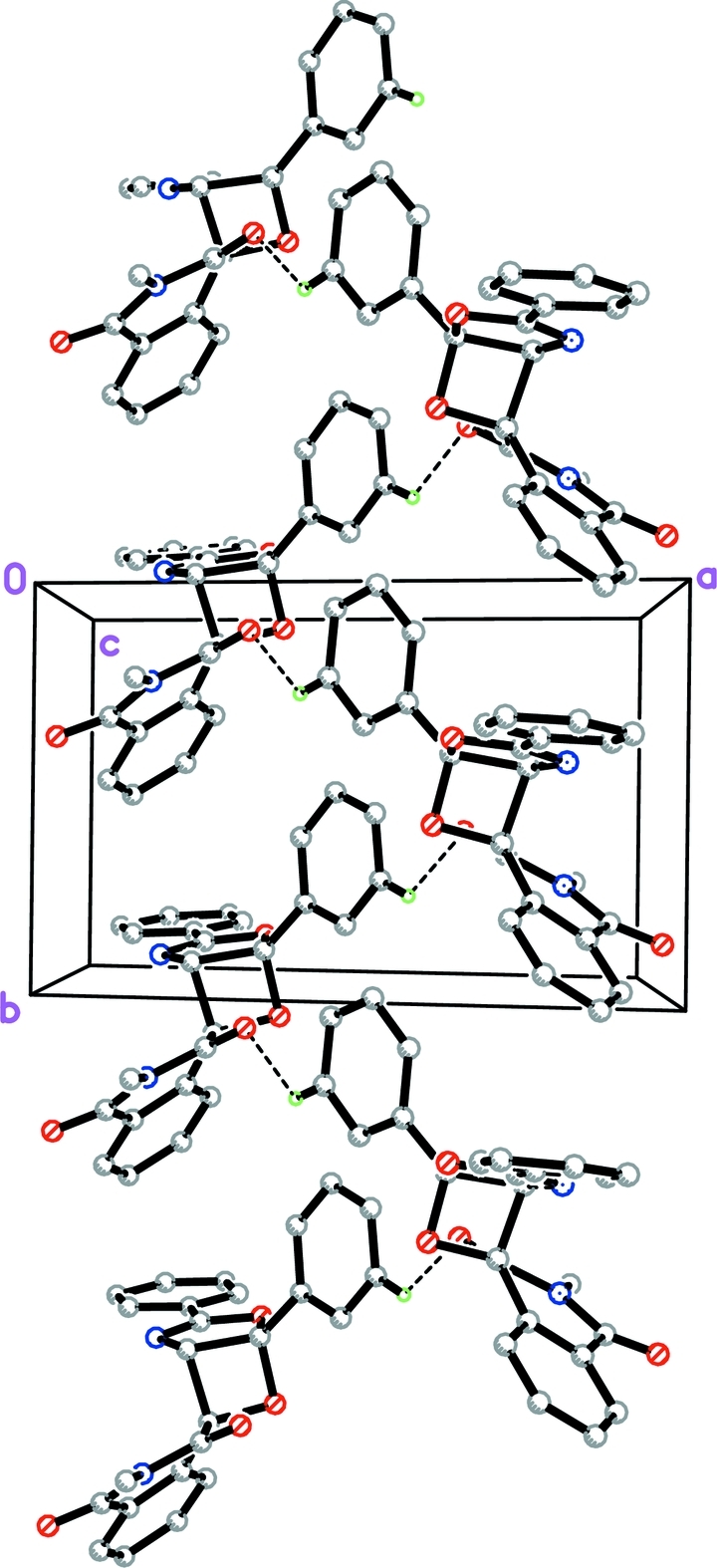
The crystal structure of the title compound, viewed along the c axis, showing a hydrogen-bonded (dashed lines) chain along the b axis. H atoms not involved in the interactions have been omitted for clarity.

## supplementary crystallographic information

### Comment

1,3,4(2*H*)-Isoquinolinetrione derivatives have a variety of biological activities and are synthetic precursors for many naturally occuring alkaloids. On the other hand, the *N*-analogues of homophthalic anhydride, 1,3-isoquinolinedione and its derivatives, have a wide range of biological activities and their structural modifications with the aim of finding new drugs and medicine have drawn increasing research interests (Malamas & Hohman, 1994; Hall *et al.*, 1994). Some substituted 1,3,4(2*H*)-isoquinolinetrione and their derivatives have been reported to treat neurodegenerative diseases, especially as the medicine for Alzhermer's disease, apoplexy and brain ischernic injuries (Nan *et al.*, 2004). The crystal structure of Z-2-methyl-3'-phenyl-spiro[isoquinoline-4,2'-oxirane]-1,3-dione has been reported (Wang *et al.*, 2000). In view of the importance of the title compound as a caspase inhibitor, this paper reports its crystal structure.

In the title isoquinoline-1,3-dione derivative (Fig. 1), the dihydro-oxazole ring (C10/C11/O4/C12/N2) is essentially planar, with a maximum deviation of 0.019 (1) Å at atom O4. The oxazole ring makes dihedral angles of 73.43 (8) and 4.24 (8)°, respectively, with C13–C18 and C19–C24 benzene rings attached to it. The tetrahydropyridine ring of the tetrahydroisoquinoline ring system adopts a distorted envelope conformation with spiro carbon C9 as the flap; the puckering amplitude Q = 0.352 (2) Å; θ = 108.2 (3)° and φ = 287.0 (3)° (Cremer & Pople, 1975). The oxetane plane (C9-C11/O3) is inclined at a dihedral angle of 67.44 (9)° with the oxazole ring. Bond lengths (Allen *et al.*, 1987) and angles are normal and comparable to those observed in related isoquinoline-1,3-dione structures (Fun *et al.*, 2010; Wang *et al.*, 2000).

In the crystal structure (Fig. 2), intermolecular C17—H17A···O1 hydrogen bonds (Table 1) link the molecules into zigzag chains along the *b* axis. The adjacent chains are cross-linked by intermolecular C—H···π interactions (Table 1) involving the C13-C18 benzene ring (centroid Cg1).

### Experimental

The title compound was obtained from the reaction between 1,3,4(2*H*)-isoquinolinetrione (189 mg, 1 mmol) and 2,5-diphenyloxazole (440 mg, 2 mmol). The compound was purified by flash column chromatography in ethyl acetate and petroleum ether. X-ray quality single crystals of the title compound were obtained by slow evaporation of a chloroform solution.

### Refinement

All the H atoms were located in a difference Fourier map [C–H = 0.95 (2)–1.021 (18) Å] and allowed to refine freely. The highest residual electron density peak is located at 0.75 Å from H10A and the deepest hole is located at 0.86 Å from C18.

### Crystal data

**Table:**

C_25_H_18_N_2_O_4_	*F*(000) = 856
*M**_r_* = 410.41	*D*_x_ = 1.412 Mg m^−^^3^
Monoclinic, *P*2_1_/*c*	Cu *K*α radiation, λ = 1.54178 Å
Hall symbol: -P 2ybc	Cell parameters from 9894 reflections
*a* = 13.3142 (3) Å	θ = 3.5–64.6°
*b* = 8.0366 (2) Å	µ = 0.79 mm^−^^1^
*c* = 19.1913 (5) Å	*T* = 100 K
β = 109.882 (1)°	Block, colourless
*V* = 1931.09 (8) Å^3^	0.27 × 0.26 × 0.25 mm
*Z* = 4	

### Data collection

**Table:**

Bruker SMART APEX DUO CCD area-detector diffractometer	3254 independent reflections
Radiation source: fine-focus sealed tube	3227 reflections with *I* > 2σ(*I*)
none	*R*_int_ = 0.018
φ and ω scans	θ_max_ = 65.0°, θ_min_ = 3.5°
Absorption correction: multi-scan (*SADABS*; Bruker, 2009)	*h* = −15→15
*T*_min_ = 0.815, *T*_max_ = 0.830	*k* = −9→8
32042 measured reflections	*l* = −22→22

### Refinement

**Table:**

Refinement on *F*^2^	Secondary atom site location: difference Fourier map
Least-squares matrix: full	Hydrogen site location: inferred from neighbouring sites
*R*[*F*^2^ > 2σ(*F*^2^)] = 0.047	All H-atom parameters refined
*wR*(*F*^2^) = 0.150	*w* = 1/[σ^2^(*F*_o_^2^) + (0.0926*P*)^2^ + 0.3391*P*] where *P* = (*F*_o_^2^ + 2*F*_c_^2^)/3
*S* = 1.35	(Δ/σ)_max_ = 0.001
3254 reflections	Δρ_max_ = 0.80 e Å^−^^3^
353 parameters	Δρ_min_ = −0.88 e Å^−^^3^
0 restraints	Extinction correction: *SHELXTL* (Sheldrick, 2008), Fc^*^=kFc[1+0.001xFc^2^λ^3^/sin(2θ)]^-1/4^
Primary atom site location: structure-invariant direct methods	Extinction coefficient: 0.052 (3)

### Special details

**Table:**

Experimental. The crystal was placed in the cold stream of an Oxford Cryosystems Cobra open-flow nitrogen cryostat (Cosier & Glazer, 1986) operating at 100.0 (1)K.
Geometry. All esds (except the esd in the dihedral angle between two l.s. planes) are estimated using the full covariance matrix. The cell esds are taken into account individually in the estimation of esds in distances, angles and torsion angles; correlations between esds in cell parameters are only used when they are defined by crystal symmetry. An approximate (isotropic) treatment of cell esds is used for estimating esds involving l.s. planes.
Refinement. Refinement of F^2^ against ALL reflections. The weighted R-factor wR and goodness of fit S are based on F^2^, conventional R-factors R are based on F, with F set to zero for negative F^2^. The threshold expression of F^2^ > 2sigma(F^2^) is used only for calculating R-factors(gt) etc. and is not relevant to the choice of reflections for refinement. R-factors based on F^2^ are statistically about twice as large as those based on F, and R- factors based on ALL data will be even larger.

### Fractional atomic coordinates and isotropic or equivalent isotropic displacement parameters (Å^2^)

**Table:**

	*x*	*y*	*z*	*U*_iso_*/*U*_eq_	
O1	0.67405 (9)	0.09357 (15)	0.23596 (6)	0.0292 (3)	
O2	0.98657 (9)	0.36419 (16)	0.27093 (6)	0.0317 (3)	
O3	0.62301 (8)	0.07638 (12)	0.08429 (5)	0.0200 (3)	
O4	0.65067 (8)	−0.12787 (13)	0.00309 (5)	0.0197 (3)	
N1	0.83241 (10)	0.22292 (16)	0.25592 (7)	0.0219 (3)	
N2	0.82562 (9)	−0.07527 (14)	0.06914 (6)	0.0185 (3)	
C1	0.73931 (12)	0.14769 (18)	0.21089 (8)	0.0210 (4)	
C2	0.90196 (12)	0.31220 (19)	0.22920 (8)	0.0225 (4)	
C3	0.86313 (11)	0.34634 (18)	0.14836 (8)	0.0206 (4)	
C4	0.91327 (12)	0.4707 (2)	0.12193 (9)	0.0253 (4)	
C5	0.87765 (13)	0.5082 (2)	0.04699 (9)	0.0265 (4)	
C6	0.79084 (13)	0.42300 (19)	−0.00134 (9)	0.0244 (4)	
C7	0.74074 (12)	0.29813 (19)	0.02473 (8)	0.0216 (4)	
C8	0.77731 (11)	0.25907 (18)	0.09976 (8)	0.0187 (4)	
C9	0.73081 (11)	0.11985 (18)	0.13037 (8)	0.0190 (4)	
C10	0.76955 (11)	−0.06240 (18)	0.12152 (8)	0.0182 (4)	
C11	0.65008 (12)	−0.09381 (18)	0.07673 (8)	0.0193 (4)	
C12	0.75508 (11)	−0.10798 (17)	0.00665 (8)	0.0182 (4)	
C13	0.58169 (11)	−0.21713 (18)	0.09854 (8)	0.0194 (4)	
C14	0.59034 (13)	−0.3863 (2)	0.08529 (9)	0.0247 (4)	
C15	0.53327 (13)	−0.5023 (2)	0.11064 (9)	0.0278 (4)	
C16	0.46778 (12)	−0.4499 (2)	0.14951 (9)	0.0263 (4)	
C17	0.45793 (12)	−0.2815 (2)	0.16149 (8)	0.0246 (4)	
C18	0.51465 (11)	−0.1653 (2)	0.13609 (8)	0.0219 (4)	
C19	0.77737 (12)	−0.12908 (17)	−0.06280 (8)	0.0192 (4)	
C20	0.88379 (12)	−0.12115 (18)	−0.05944 (9)	0.0215 (4)	
C21	0.90942 (13)	−0.13662 (19)	−0.12331 (9)	0.0259 (4)	
C22	0.82889 (14)	−0.1618 (2)	−0.19087 (9)	0.0286 (4)	
C23	0.72301 (13)	−0.1716 (2)	−0.19467 (9)	0.0284 (4)	
C24	0.69684 (12)	−0.15463 (18)	−0.13063 (8)	0.0229 (4)	
C25	0.85260 (14)	0.2178 (2)	0.33612 (8)	0.0267 (4)	
H4A	0.9730 (17)	0.530 (3)	0.1601 (11)	0.038 (5)*	
H5A	0.9128 (16)	0.594 (3)	0.0298 (11)	0.030 (5)*	
H6A	0.7619 (14)	0.453 (2)	−0.0564 (10)	0.023 (4)*	
H7A	0.6790 (15)	0.241 (2)	−0.0100 (10)	0.027 (4)*	
H10A	0.8042 (13)	−0.122 (2)	0.1680 (10)	0.017 (4)*	
H14A	0.6366 (16)	−0.421 (3)	0.0580 (11)	0.036 (5)*	
H15A	0.5374 (16)	−0.622 (3)	0.0998 (11)	0.036 (5)*	
H16A	0.4261 (15)	−0.531 (3)	0.1681 (10)	0.030 (5)*	
H17A	0.4124 (15)	−0.243 (2)	0.1881 (10)	0.025 (4)*	
H18A	0.5091 (14)	−0.049 (3)	0.1448 (10)	0.025 (4)*	
H20A	0.9383 (15)	−0.105 (2)	−0.0121 (10)	0.022 (4)*	
H21A	0.9834 (15)	−0.124 (2)	−0.1203 (9)	0.023 (4)*	
H22A	0.8479 (17)	−0.174 (3)	−0.2372 (12)	0.042 (6)*	
H23A	0.6682 (16)	−0.191 (3)	−0.2414 (11)	0.031 (5)*	
H24A	0.6219 (16)	−0.159 (2)	−0.1330 (10)	0.029 (5)*	
H25A	0.7977 (15)	0.286 (2)	0.3470 (10)	0.027 (4)*	
H25B	0.8482 (14)	0.103 (3)	0.3493 (10)	0.028 (5)*	
H25C	0.9243 (16)	0.267 (2)	0.3603 (11)	0.030 (5)*	

### Atomic displacement parameters (Å^2^)

**Table:**

	*U*^11^	*U*^22^	*U*^33^	*U*^12^	*U*^13^	*U*^23^
O1	0.0334 (7)	0.0323 (7)	0.0290 (6)	−0.0065 (5)	0.0198 (5)	−0.0034 (5)
O2	0.0214 (6)	0.0460 (8)	0.0256 (6)	−0.0050 (5)	0.0051 (5)	−0.0069 (5)
O3	0.0174 (6)	0.0182 (6)	0.0238 (6)	−0.0004 (4)	0.0060 (4)	−0.0003 (4)
O4	0.0182 (6)	0.0235 (6)	0.0182 (5)	−0.0023 (4)	0.0074 (4)	−0.0009 (4)
N1	0.0231 (7)	0.0252 (7)	0.0186 (6)	0.0023 (5)	0.0085 (5)	−0.0009 (5)
N2	0.0191 (6)	0.0170 (6)	0.0201 (6)	0.0012 (4)	0.0076 (5)	0.0001 (5)
C1	0.0239 (8)	0.0185 (8)	0.0226 (8)	0.0023 (6)	0.0107 (6)	0.0005 (6)
C2	0.0205 (8)	0.0240 (8)	0.0237 (8)	0.0023 (6)	0.0085 (6)	−0.0035 (6)
C3	0.0201 (7)	0.0204 (8)	0.0232 (8)	0.0018 (6)	0.0099 (6)	−0.0028 (6)
C4	0.0229 (8)	0.0223 (8)	0.0323 (9)	−0.0032 (6)	0.0117 (7)	−0.0030 (6)
C5	0.0290 (8)	0.0207 (8)	0.0346 (9)	0.0006 (6)	0.0172 (7)	0.0047 (6)
C6	0.0285 (8)	0.0227 (8)	0.0255 (8)	0.0051 (6)	0.0138 (7)	0.0038 (6)
C7	0.0225 (8)	0.0209 (8)	0.0226 (8)	0.0016 (6)	0.0092 (6)	−0.0012 (6)
C8	0.0185 (7)	0.0172 (7)	0.0219 (7)	0.0021 (5)	0.0090 (6)	−0.0008 (5)
C9	0.0178 (7)	0.0192 (7)	0.0209 (7)	0.0002 (5)	0.0077 (6)	−0.0009 (5)
C10	0.0191 (7)	0.0181 (7)	0.0182 (7)	−0.0002 (5)	0.0073 (6)	0.0006 (5)
C11	0.0207 (8)	0.0192 (7)	0.0184 (7)	0.0012 (6)	0.0074 (6)	0.0003 (5)
C12	0.0179 (7)	0.0145 (7)	0.0228 (8)	0.0002 (5)	0.0076 (6)	0.0011 (5)
C13	0.0165 (7)	0.0216 (8)	0.0188 (7)	−0.0017 (6)	0.0045 (6)	0.0008 (6)
C14	0.0260 (8)	0.0246 (8)	0.0265 (8)	−0.0016 (6)	0.0130 (7)	−0.0026 (6)
C15	0.0311 (9)	0.0211 (8)	0.0321 (9)	−0.0044 (6)	0.0116 (7)	−0.0007 (6)
C16	0.0237 (8)	0.0283 (9)	0.0270 (8)	−0.0067 (6)	0.0085 (6)	0.0033 (6)
C17	0.0187 (7)	0.0312 (9)	0.0253 (8)	−0.0020 (6)	0.0095 (6)	0.0011 (6)
C18	0.0188 (7)	0.0226 (8)	0.0242 (8)	0.0005 (6)	0.0071 (6)	0.0006 (6)
C19	0.0231 (8)	0.0137 (7)	0.0217 (8)	−0.0001 (5)	0.0089 (6)	0.0002 (5)
C20	0.0222 (8)	0.0196 (8)	0.0229 (8)	−0.0010 (6)	0.0078 (6)	0.0002 (6)
C21	0.0269 (9)	0.0239 (8)	0.0316 (9)	−0.0013 (6)	0.0158 (7)	−0.0024 (6)
C22	0.0371 (9)	0.0284 (9)	0.0253 (8)	−0.0032 (7)	0.0172 (7)	−0.0053 (6)
C23	0.0310 (9)	0.0319 (9)	0.0213 (8)	−0.0032 (7)	0.0074 (7)	−0.0059 (6)
C24	0.0226 (8)	0.0216 (8)	0.0246 (8)	−0.0006 (6)	0.0081 (6)	−0.0018 (6)
C25	0.0304 (9)	0.0319 (10)	0.0190 (8)	0.0065 (7)	0.0100 (7)	−0.0008 (6)

### Geometric parameters (Å, °)

**Table:**

O1—C1	1.2083 (18)	C11—C13	1.499 (2)
O2—C2	1.2130 (19)	C12—C19	1.470 (2)
O3—C11	1.4343 (17)	C13—C18	1.388 (2)
O3—C9	1.4495 (17)	C13—C14	1.395 (2)
O4—C12	1.3777 (17)	C14—C15	1.391 (2)
O4—C11	1.4421 (16)	C14—H14A	0.98 (2)
N1—C1	1.386 (2)	C15—C16	1.392 (2)
N1—C2	1.399 (2)	C15—H15A	0.99 (2)
N1—C25	1.4702 (18)	C16—C17	1.386 (2)
N2—C12	1.2741 (19)	C16—H16A	1.00 (2)
N2—C10	1.4453 (18)	C17—C18	1.389 (2)
C1—C9	1.5269 (19)	C17—H17A	0.966 (18)
C2—C3	1.485 (2)	C18—H18A	0.96 (2)
C3—C4	1.390 (2)	C19—C24	1.392 (2)
C3—C8	1.394 (2)	C19—C20	1.398 (2)
C4—C5	1.386 (2)	C20—C21	1.385 (2)
C4—H4A	1.00 (2)	C20—H20A	0.960 (19)
C5—C6	1.391 (2)	C21—C22	1.388 (2)
C5—H5A	0.95 (2)	C21—H21A	0.972 (19)
C6—C7	1.389 (2)	C22—C23	1.389 (2)
C6—H6A	1.021 (18)	C22—H22A	1.01 (2)
C7—C8	1.390 (2)	C23—C24	1.393 (2)
C7—H7A	0.979 (19)	C23—H23A	0.96 (2)
C8—C9	1.4925 (19)	C24—H24A	0.98 (2)
C9—C10	1.5810 (19)	C25—H25A	0.99 (2)
C10—C11	1.550 (2)	C25—H25B	0.97 (2)
C10—H10A	0.979 (17)	C25—H25C	0.99 (2)
			
C11—O3—C9	93.49 (10)	C13—C11—C10	124.00 (12)
C12—O4—C11	105.73 (10)	N2—C12—O4	118.61 (12)
C1—N1—C2	123.94 (12)	N2—C12—C19	124.45 (13)
C1—N1—C25	116.55 (12)	O4—C12—C19	116.93 (12)
C2—N1—C25	119.32 (12)	C18—C13—C14	119.65 (14)
C12—N2—C10	106.25 (12)	C18—C13—C11	120.36 (13)
O1—C1—N1	121.37 (14)	C14—C13—C11	119.86 (13)
O1—C1—C9	122.31 (14)	C15—C14—C13	120.04 (14)
N1—C1—C9	115.96 (12)	C15—C14—H14A	120.9 (12)
O2—C2—N1	121.09 (14)	C13—C14—H14A	119.0 (12)
O2—C2—C3	122.78 (14)	C14—C15—C16	120.06 (15)
N1—C2—C3	116.04 (13)	C14—C15—H15A	120.1 (12)
C4—C3—C8	120.25 (14)	C16—C15—H15A	119.8 (12)
C4—C3—C2	118.33 (14)	C17—C16—C15	119.76 (14)
C8—C3—C2	121.42 (13)	C17—C16—H16A	119.0 (11)
C5—C4—C3	119.85 (14)	C15—C16—H16A	121.2 (11)
C5—C4—H4A	124.3 (12)	C16—C17—C18	120.30 (14)
C3—C4—H4A	115.8 (12)	C16—C17—H17A	120.8 (11)
C4—C5—C6	119.90 (14)	C18—C17—H17A	118.9 (11)
C4—C5—H5A	118.7 (12)	C13—C18—C17	120.17 (14)
C6—C5—H5A	121.4 (12)	C13—C18—H18A	119.1 (11)
C7—C6—C5	120.48 (14)	C17—C18—H18A	120.8 (11)
C7—C6—H6A	118.8 (10)	C24—C19—C20	119.76 (14)
C5—C6—H6A	120.7 (10)	C24—C19—C12	122.41 (13)
C6—C7—C8	119.63 (14)	C20—C19—C12	117.82 (13)
C6—C7—H7A	119.3 (11)	C21—C20—C19	120.39 (15)
C8—C7—H7A	121.1 (11)	C21—C20—H20A	121.0 (11)
C7—C8—C3	119.88 (13)	C19—C20—H20A	118.6 (11)
C7—C8—C9	122.08 (13)	C20—C21—C22	119.69 (15)
C3—C8—C9	117.98 (13)	C20—C21—H21A	119.2 (10)
O3—C9—C8	113.43 (11)	C22—C21—H21A	121.0 (10)
O3—C9—C1	111.72 (11)	C21—C22—C23	120.34 (15)
C8—C9—C1	112.99 (12)	C21—C22—H22A	119.5 (12)
O3—C9—C10	90.15 (10)	C23—C22—H22A	120.2 (12)
C8—C9—C10	117.26 (11)	C22—C23—C24	120.14 (15)
C1—C9—C10	109.26 (11)	C22—C23—H23A	119.7 (11)
N2—C10—C11	106.14 (11)	C24—C23—H23A	120.2 (11)
N2—C10—C9	113.93 (11)	C19—C24—C23	119.68 (14)
C11—C10—C9	84.25 (10)	C19—C24—H24A	119.7 (11)
N2—C10—H10A	113.8 (10)	C23—C24—H24A	120.6 (11)
C11—C10—H10A	120.1 (10)	N1—C25—H25A	108.7 (11)
C9—C10—H10A	115.3 (10)	N1—C25—H25B	107.2 (11)
O3—C11—O4	111.43 (11)	H25A—C25—H25B	110.9 (15)
O3—C11—C13	113.89 (12)	N1—C25—H25C	106.5 (11)
O4—C11—C13	110.76 (11)	H25A—C25—H25C	110.3 (15)
O3—C11—C10	91.97 (10)	H25B—C25—H25C	113.0 (16)
O4—C11—C10	103.17 (11)		
			
C2—N1—C1—O1	−168.82 (14)	C8—C9—C10—C11	−119.42 (13)
C25—N1—C1—O1	6.1 (2)	C1—C9—C10—C11	110.37 (12)
C2—N1—C1—C9	18.0 (2)	C9—O3—C11—O4	−108.01 (12)
C25—N1—C1—C9	−167.07 (13)	C9—O3—C11—C13	125.81 (12)
C1—N1—C2—O2	−174.06 (14)	C9—O3—C11—C10	−3.03 (10)
C25—N1—C2—O2	11.1 (2)	C12—O4—C11—O3	94.44 (12)
C1—N1—C2—C3	9.2 (2)	C12—O4—C11—C13	−137.68 (12)
C25—N1—C2—C3	−165.64 (13)	C12—O4—C11—C10	−3.02 (14)
O2—C2—C3—C4	−14.3 (2)	N2—C10—C11—O3	−110.49 (11)
N1—C2—C3—C4	162.41 (13)	C9—C10—C11—O3	2.79 (9)
O2—C2—C3—C8	166.92 (14)	N2—C10—C11—O4	2.06 (14)
N1—C2—C3—C8	−16.4 (2)	C9—C10—C11—O4	115.34 (11)
C8—C3—C4—C5	0.1 (2)	N2—C10—C11—C13	128.72 (14)
C2—C3—C4—C5	−178.67 (13)	C9—C10—C11—C13	−118.01 (14)
C3—C4—C5—C6	1.0 (2)	C10—N2—C12—O4	−2.00 (16)
C4—C5—C6—C7	−1.3 (2)	C10—N2—C12—C19	179.06 (13)
C5—C6—C7—C8	0.5 (2)	C11—O4—C12—N2	3.43 (17)
C6—C7—C8—C3	0.6 (2)	C11—O4—C12—C19	−177.55 (11)
C6—C7—C8—C9	−176.45 (13)	O3—C11—C13—C18	−12.07 (19)
C4—C3—C8—C7	−0.9 (2)	O4—C11—C13—C18	−138.61 (13)
C2—C3—C8—C7	177.82 (13)	C10—C11—C13—C18	98.05 (17)
C4—C3—C8—C9	176.28 (13)	O3—C11—C13—C14	172.03 (13)
C2—C3—C8—C9	−5.0 (2)	O4—C11—C13—C14	45.49 (17)
C11—O3—C9—C8	122.99 (12)	C10—C11—C13—C14	−77.85 (19)
C11—O3—C9—C1	−107.89 (12)	C18—C13—C14—C15	−1.0 (2)
C11—O3—C9—C10	2.97 (10)	C11—C13—C14—C15	174.94 (13)
C7—C8—C9—O3	−23.27 (19)	C13—C14—C15—C16	−0.3 (2)
C3—C8—C9—O3	159.58 (12)	C14—C15—C16—C17	1.4 (2)
C7—C8—C9—C1	−151.74 (13)	C15—C16—C17—C18	−1.2 (2)
C3—C8—C9—C1	31.12 (17)	C14—C13—C18—C17	1.1 (2)
C7—C8—C9—C10	79.82 (17)	C11—C13—C18—C17	−174.77 (12)
C3—C8—C9—C10	−97.33 (15)	C16—C17—C18—C13	0.0 (2)
O1—C1—C9—O3	19.88 (19)	N2—C12—C19—C24	−175.95 (14)
N1—C1—C9—O3	−166.99 (12)	O4—C12—C19—C24	5.1 (2)
O1—C1—C9—C8	149.23 (14)	N2—C12—C19—C20	3.4 (2)
N1—C1—C9—C8	−37.65 (17)	O4—C12—C19—C20	−175.55 (12)
O1—C1—C9—C10	−78.29 (17)	C24—C19—C20—C21	0.8 (2)
N1—C1—C9—C10	94.83 (14)	C12—C19—C20—C21	−178.63 (13)
C12—N2—C10—C11	−0.22 (14)	C19—C20—C21—C22	−0.7 (2)
C12—N2—C10—C9	−91.04 (14)	C20—C21—C22—C23	0.0 (2)
O3—C9—C10—N2	102.37 (12)	C21—C22—C23—C24	0.6 (2)
C8—C9—C10—N2	−14.29 (18)	C20—C19—C24—C23	−0.2 (2)
C1—C9—C10—N2	−144.50 (12)	C12—C19—C24—C23	179.18 (14)
O3—C9—C10—C11	−2.76 (9)	C22—C23—C24—C19	−0.5 (2)

### Hydrogen-bond geometry (Å, °)

**Table:**

Cg1 is the centroid of the C13–C18 benzene ring.

**Table:**

*D*—H···*A*	*D*—H	H···*A*	*D*···*A*	*D*—H···*A*
C17—H17A···O1^i^	0.97 (2)	2.51 (2)	3.213 (2)	129 (1)
C23—H23A···Cg1^ii^	0.96 (2)	2.66 (2)	3.5904 (18)	166 (2)

Symmetry codes: (i) −*x*+1, *y*−1/2, −*z*+1/2; (ii) *x*, −*y*−3/2, *z*−3/2.

## References

[bb1] Allen, F. H., Kennard, O., Watson, D. G., Brammer, L., Orpen, A. G. & Taylor, R. (1987). *J. Chem. Soc. Perkin Trans. 2*, pp. S1–19.

[bb2] Bruker (2009). *APEX2*, *SAINT* and *SADABS* Bruker AXS Inc., Madison, Wisconsin, USA.

[bb3] Cosier, J. & Glazer, A. M. (1986). *J. Appl. Cryst.* **19**, 105–107.

[bb4] Cremer, D. & Pople, J. A. (1975). *J. Am. Chem. Soc.* **97**, 1354–1358.

[bb5] Fun, H.-K., Goh, J. H., Yu, H. & Zhang, Y. (2010). *Acta Cryst.* E**66**, o724–o725. 10.1107/S1600536810007397 PMC2983926 21580572

[bb6] Hall, I. H., Chapman, J. M. & Wong, O. T. (1994). *Anticancer Drugs*, **5**, 75–82. 10.1097/00001813-199402000-00012 8186434

[bb7] Malamas, M. S. & Hohman, T. C. (1994). *J. Med. Chem.* **37**, 2043–2058. 10.1021/jm00039a017 8027986

[bb8] Nan, F. J., Li, J., Chen, Y. H., Zhang, Y. H., Gu, M. & Zhang, H. J. (2004). PCT Int. Appl. WO 2004111010 A1.

[bb9] Sheldrick, G. M. (2008). *Acta Cryst.* A**64**, 112–122. 10.1107/S0108767307043930 18156677

[bb10] Spek, A. L. (2009). *Acta Cryst.* D**65**, 148–155. 10.1107/S090744490804362X PMC2631630 19171970

[bb11] Wang, X.-L., Tian, J.-Z., Ling, K.-Q. & Xu, J.-H. (2000). *Res. Chem. Intermed.* **26**, 679–689.

